# Ability of the COPD Assessment Test to evaluate the lung‐specific quality of life in systemic sclerosis‐associated interstitial lung disease

**DOI:** 10.1111/crj.13162

**Published:** 2020-02-19

**Authors:** Naoki Mugii, Fujiko Someya

**Affiliations:** ^1^ Graduate School of Health Sciences, College of Medical, Pharmaceutical and Health Sciences Kanazawa University Kanazawa Japan; ^2^ Kanazawa University Hospital Kanazawa University Kanazawa Japan; ^3^ School of Health Sciences Kanazawa University Kanazawa Japan

**Keywords:** exercise capacity, interstitial lung disease, pulmonary arterial hypertension, pulmonary function, quality of life, systemic sclerosis

## Abstract

**Introduction:**

The COPD Assessment Test (CAT) is utilised to evaluate the treatment outcome regarding the health status in idiopathic interstitial pneumonia (IIP). However, the ability of the CAT to evaluate the lung‐specific quality of life in systemic sclerosis‐associated interstitial lung disease (SSc‐ILD) is not established. Therefore, we investigated whether CAT scores can be used to evaluate SSc‐ILD as they are for IIP.

**Methods:**

A total of 150 patients with IIP or SSc‐ILD who were evaluated by the CAT were retrospectively assigned to this study. Clinical data at the visit for the CAT were analysed.

**Results:**

The forced vital capacity and distance walked during the 6‐minute walk test (6MWD) were significantly correlated with the CAT score for SSc‐ILD and IIP, and the CAT scores were similarly distributed in SSc‐ILD and IIP. The CAT score of SSc‐ILD patients was negatively affected by pulmonary arterial hypertension, but not by corticosteroids, which affected it in IIP patients. CAT scores of patients with either disease receiving home oxygen therapy were poor. In multiple regression analysis, pulmonary arterial hypertension and 6MWD were independent predictors for the CAT score in patients with SSc‐ILD, while corticosteroid administration was selected as an independent factor in patients with IIP.

**Conclusions:**

Our study suggests that the CAT can be applied to evaluate the lung‐specific quality of life in SSc‐ILD similar to IIP regarding the pulmonary function, but it should be noted that pulmonary arterial hypertension in SSc‐ILD influences the CAT score.

## 1 INTRODUCTION

Evaluation of the health‐related quality of life is valuable for the comprehensive assessment of patients with progressive disease and the COPD Assessment Test (CAT) is a useful and simple evaluation tool for the lung‐specific quality of life for chronic obstructive pulmonary disease (COPD) patients.^1^, ^2^, ^3^ Effective treatment for COPD patients can be administered when an improvement in the CAT score is observed,^4^, ^5^, ^6^ which implies that the CAT is an important evaluation for treatment strategies. Recently, the CAT has also been utilised to evaluate the health status of patients with idiopathic interstitial pneumonia (IIP).^7^ As for idiopathic pulmonary fibrosis, which primarily impairs the lung, the CAT score is correlated with the percentage of predicted forced vital capacity (FVC), the diffusion capacity of the lung for carbon monoxide (DLCO) and distance walked during the 6‐minute walk test (6MWD).^8^

Connective tissue disease‐associated interstitial lung disease (CTD‐ILD) patients were also evaluated by the CAT and it was reported to be significantly associated with the measures of pulmonary function, 6MWD and the Medical Research Council scale.^9^ However, CTDs include many diseases and it is unclear whether the CAT scores in CTD‐ILD are similar to those in IIP in terms of pulmonary involvement because no study has compared the CAT scores in CTD‐ILD with those in IIP. Among CTDs, systemic sclerosis (SSc) prominently involves the skin tissue‐related impairment of joint motion and the Health Assessment Questionnaire (HAQ) is commonly used to measure the quality of life based on physical disability in the patients.^10^ SSc patients are known to frequently develop ILD, leading to mortality.^11^ However, the items in the HAQ do not evaluate respiratory disorder and it is difficult to assess the accurate health status in terms of lung impairment in patients with systemic sclerosis‐associated interstitial lung disease (SSc‐ILD).^12^, ^13^

This study evaluated whether CAT scores in SSc‐ILD were associated with pulmonary function and 6MWD as in IIP, and examined the differences in CAT scores based on clinical interventions and complications for each disease. The FVC% predicted was used to represent the pulmonary function in this study following a previous study.^11^ In general, the percent predicted forced expiratory volume in 1 second is the most important parameter in the pulmonary function tests for classifying severity in COPD.^3^ However, the value does not easily decrease in ILD and it is known that scores evaluated by the St. George Respiratory Questionnaire were poorer for patients with ILD than for patients with COPD with a similar percent predicted forced expiratory volume in 1 second.^14^

## 2 MATERIALS AND METHODS

The patients in this study were diagnosed with ILD by high‐resolution computed tomography or lung biopsy at our facility and were transferred to the rehabilitation division for further physical treatment. We evaluated them using the CAT, and CAT scores and patient records on the first visit between January 2013 and October 2018 were collected. Sixty patients with IIP and 90 with SSc‐ILD, 150 patients in total, were assigned to this study.

As a retrospective study, the use of patient records for this study was approved by the Human Ethics Committee of Kanazawa University conforming to the provisions of the Declaration of Helsinki. Following approval, the study was publically announced on the bulletin board and the home page of our facility for patients to opt out, although written informed consent for participation in the study was not received from patients.

Demographic information, medical history, FVC% predicted and 6MWD were chosen from patient records. Corticosteroid administration, home oxygen therapy and history of pulmonary arterial hypertension (PAH) at the visit were selected as possible factors influencing the CAT score. PAH was diagnosed by heart catheterisation after screening using Doppler echocardiography.

### 2.1 Statistical analysis

For gender distribution, presentation of PAH, corticosteroids medication, and home oxygen therapy, the *χ* ^2^ test was used to compare the numbers of patients with IIP or SSc‐ILD. The age, height, weight, BMI and time since diagnosis of patients were compared using the Student’s *t* test. The time since diagnosis was the duration from the time diagnosed to the first CAT evaluation. The difference of CAT score was examined using the Mann‐Whitney test between IIP and SSc‐ILD or among the presence of corticosteroids, home oxygen therapy, and history of PAH for each disease. The relationships between the CAT score and time since diagnosis, FVC% predicted, and 6MWD were examined by the Spearman’s correlation test. The significant variables were selected from FVC% predicted, 6MWD, the presence of corticosteroids, home oxygen therapy and history of PAH for subsequent stepwise multiple regression analyses using the CAT scores in IIP and SSc‐ILD. JMP 11 software (SAS Institute Inc.) was used for statistical analysis. *P* < 0.05 was considered significant.

### 2.2 Results

The IIP patients included 12 with idiopathic pulmonary fibrosis. Autoantibodies in SSc‐ILD patients were anti‐RNA polymerase antibody in 15, anticentromere antibody in 10, anti‐topoisomerase I antibody in 52 and others, such as anti‐U1 RNP antibody, in 13. The female‐to‐male ratio was low among IIP patients, but high among those with SSc‐ILD, which may be due to disease specificity (Table 1).^15^, ^16^ Patients with IIP were taller and heavier than patients with SSc‐ILD, likely because of the difference in gender distribution. The SSc‐ILD patients showed longer disease duration than the IIP patients and there was a significant correlation between time since diagnosis and the CAT score (*r* = 0.25, *P* < 0.05). Such a correlation was not seen in the IIP patients. The lower CAT score and longer 6MWD in SSc‐ILD patients were also observed. The number of patients on corticosteroids’ medication was bigger in SSc‐ILD than in IIP.

**Table 1:** Patient demographics

	IIP N = 60	SSc‐ILD N = 90	*P*
Gender (f/m) (% female)	14/46 (23%)	71/19 (79%)	<0.01
Age (years)	69 ± 11	59 ± 14	<0.01
Height (cm)	162 ± 8	158 ± 9	<0.01
Weight (kg)	61 ± 12	52 ± 11	<0.01
BMI	23.2 ± 4.2	20.9 ± 3.3	<0.01
Time since diagnosis (years)	4.2 ± 4.8	8.2 ± 7.5	<0.01
CAT score	15 (8.25‐21.5)	11 (5‐17.25)	0.02
FVC, % predicted	81.4 ± 24.8	86.7 ± 24.5	NS
6MWD (m)	393 ± 135	463 ± 15.9	0.01
PAH (yes/no)	3/57	10/80	NS
Corticosteroids (yes/no)	15/45	45/45	<0.01
Home oxygen therapy (yes/no)	5/55	6/84	NS

Data are expressed as the mean ± SD, the median (25th‐75th percentiles) or absolute numbers.

Abbreviations: FVC, forced vital capacity; IIP, idiopathic interstitial pneumonia; PAH, pulmonary arterial hypertension; SSc‐ILD, systemic sclerosis‐associated interstitial lung disease; 6MWD, distance walked during the 6‐minute walk test.

**Figure 1:**
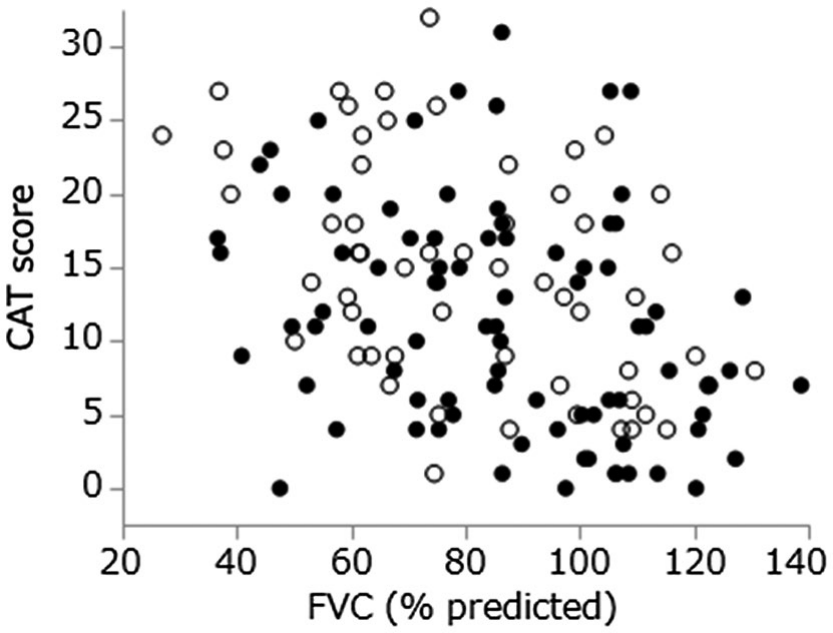
Correlation of the CAT score and forced vital capacity. Open circles, idiopathic interstitial pneumonia (*r* = −0.47, *P* < 0.01); filled circles, systemic sclerosis‐associated interstitial lung disease (*r* = −0.34, *P* < 0.01)

**Figure 2:**
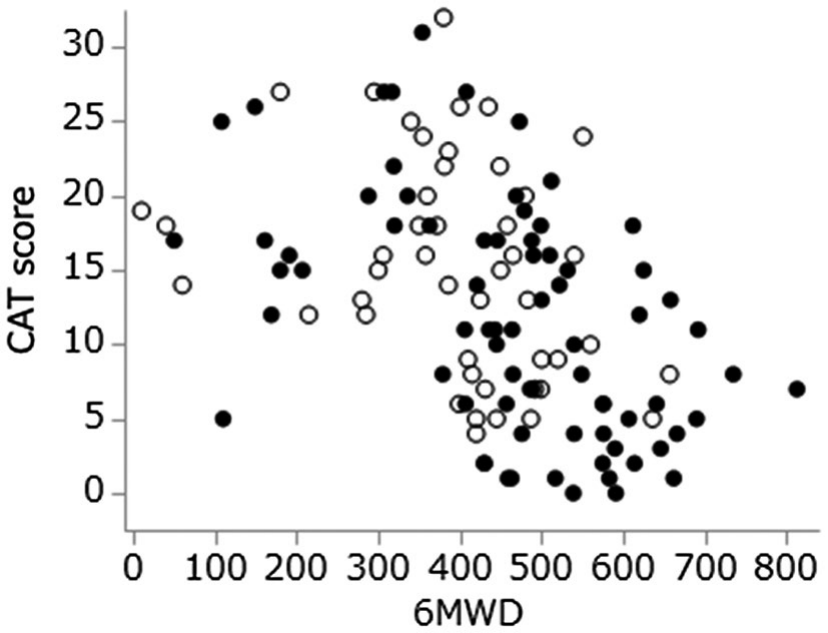
Correlation of the CAT score and distance walked during the 6‐minute walk test. Open circles, idiopathic interstitial pneumonia (*r* = −0.40, *P* < 0.01); filled circles, systemic sclerosis‐associated interstitial lung disease (*r* = −0.51, *P* < 0.01)

The CAT scores for IIP and SSc‐ILD were significantly correlated with the FVC% predicted (Figure 1) and 6MWD (Figure 2). As they were examined by the Spearman’s correlation test, no regression line is presented. It seems no difference for the relationships between the evaluation variables in IIP and SSc‐ILD, because plotted data are distributed almost in the same area.

**Figure 3:**
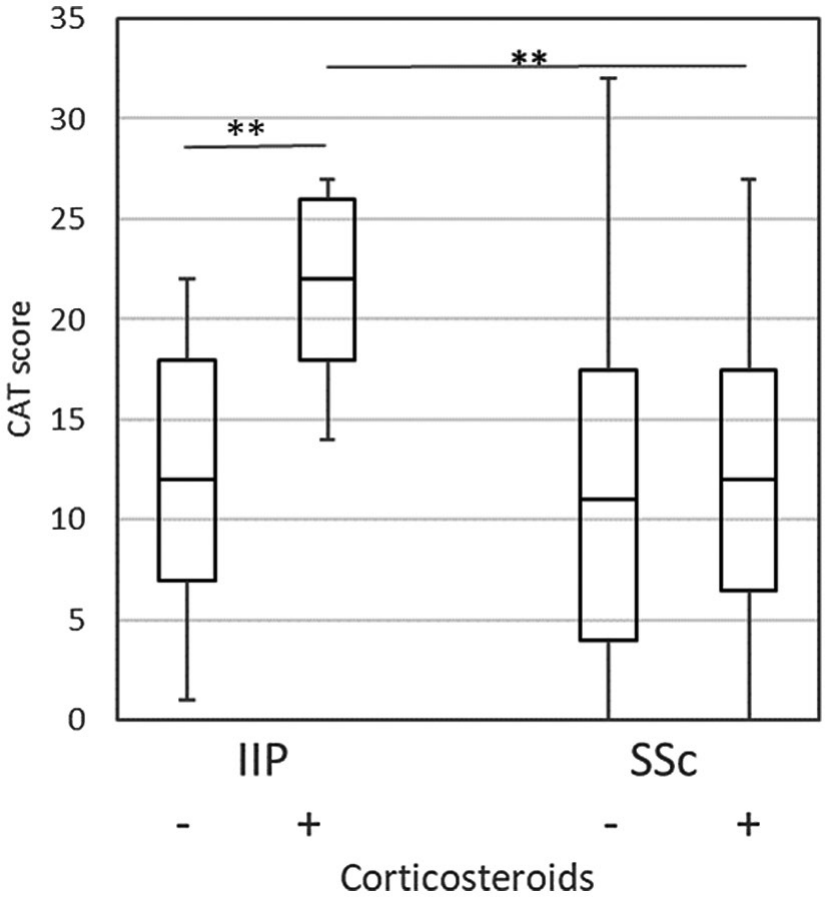
Comparison of the CAT score for corticosteroids’ medication. Boxes represent medians and interquartile ranges, and whiskers represent the lowest and highest data. **P < 0.01; IIP, idiopathic interstitial pneumonia; SSc, systemic sclerosis‐associated interstitial lung disease

**Figure 4:**
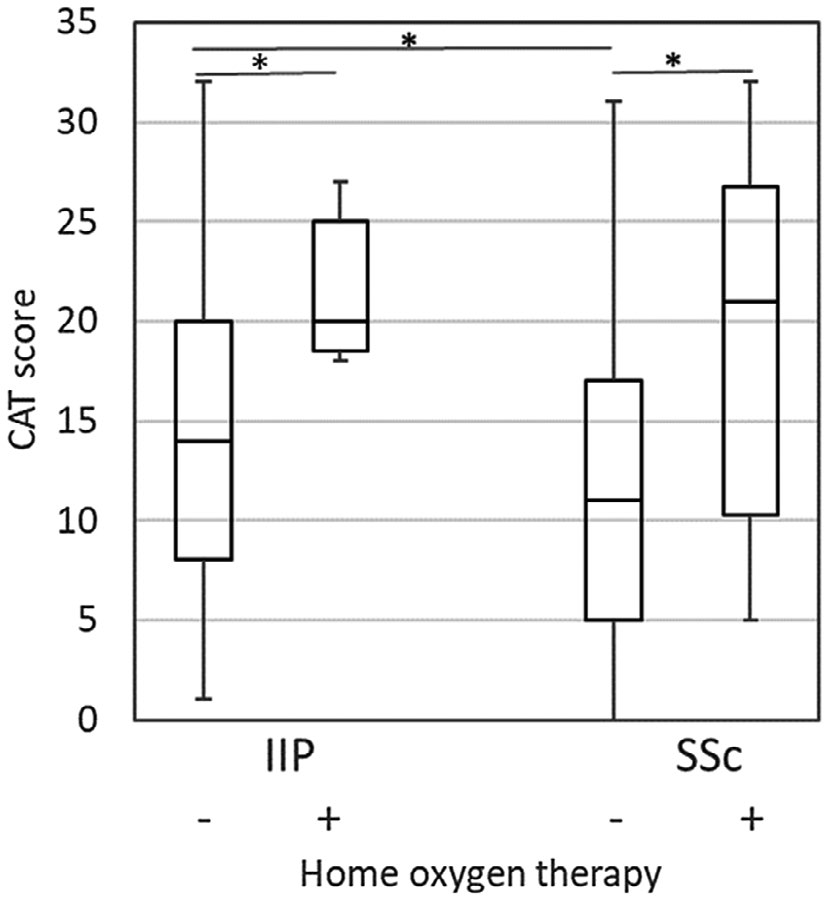
Comparison of the CAT score for home oxygen therapy. Boxes represent medians and interquartile ranges, and whiskers represent the lowest and highest data. *P < 0.05; IIP, idiopathic interstitial pneumonia; SSc, systemic sclerosis‐associated interstitial lung disease

**Figure 5:**
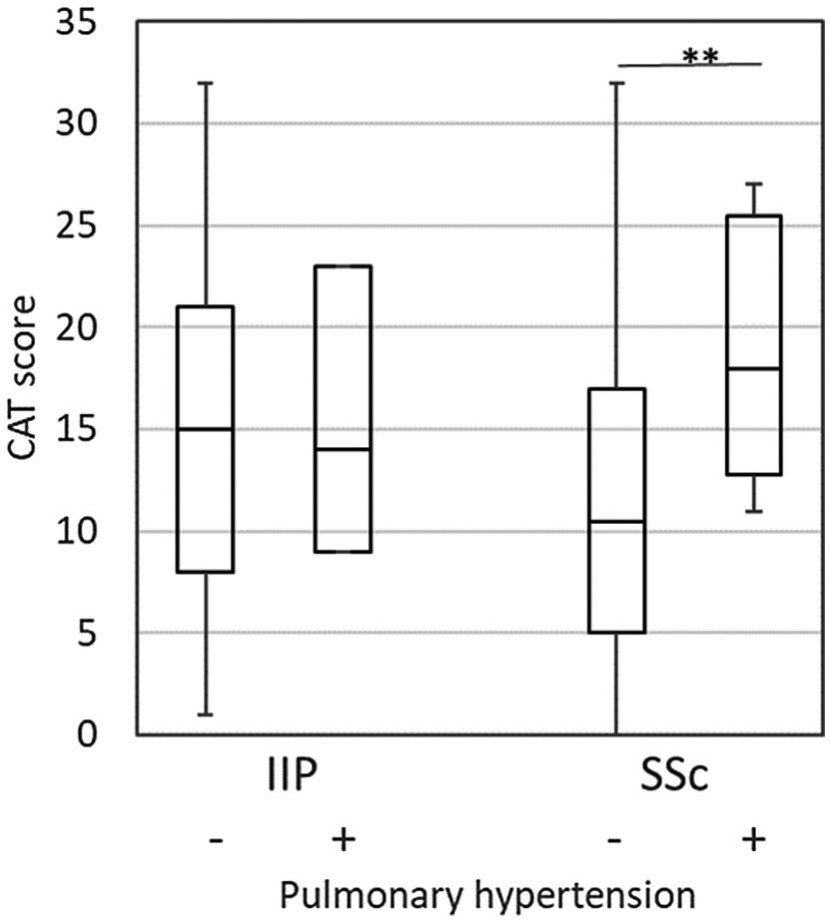
Comparison of the CAT score for pulmonary arterial hypertension. Boxes represent medians and interquartile ranges, and whiskers represent the lowest and highest data. **P < 0.01; IIP, idiopathic interstitial pneumonia; SSc, systemic sclerosis‐associated interstitial lung disease

COPD Assessment Test scores in IIP were poorer in the presence of medication with corticosteroids (Figure 3) or home oxygen therapy (Figure 4). It should be noted that the CAT score in IIP patients taking corticosteroids was significantly poorer than that in SSc‐ILD patients. In SSc‐ILD, the scores were poorer in patients with PAH (Figure 5) or home oxygen therapy, but not with corticosteroids. There were only three patients with PAH in IIP, making comparison impossible for that group.

Multiple regression analysis revealed that medication with corticosteroids was a significant factor affecting the CAT score in patients with IIP (*r* ^2^ = 0.19), while PAH and 6MWD were independent factors in patients with SSc‐ILD (*r* ^2^ = 0.31) (Table 2).

**Table 2:** Associations of CAT scores with clinically accompanying conditions by multiple regression analysis

	B	SE	95%CI	*β*	*P*	*R* ^2^
IIP						
Corticosteroids	−4.41	0.99	−6.39 to −2.44	−4.47	<0.01	0.19
SSc‐ILD						
PAH	−3.19	1.39	−5.97 to −0.41	−2.29	0.03	0.31
6MWD	−0.02	0.01	−0.03 to −0.01	−4.06	<0.01	

Abbreviations: IIP, idiopathic interstitial pneumonia; PAH, pulmonary arterial hypertension; SSc‐ILD, systemic sclerosis‐associated interstitial lung disease; 6MWD, distance walked during the 6‐minute walk test.

## 3 DISCUSSION

In this study, the CAT score for patients with IIP or SSc‐ILD was significantly correlated with the FVC% predicted and 6MWD, as found in the previous study.^7^ Other evaluation tools for the lung‐specific quality of life, such as the St. George’s Respiratory Questionnaire, the Medical Research Council scale and the Leicester Cough Questionnaire scores, were also demonstrated to be closely related to the CAT score in IIP patients.^7^ Moreover, it was reported that the CAT score related to hospital anxiety and depression scale in patients with IIP. Whereas, Lumetti et al^17^ showed that the Short Form‐36 and HAQ were not correlated with objective lung damage as assessed by spirometry and pulmonary fibrosis radiology in SSc‐ILD. It suggests that the CAT is a valid measurement for assessing the lung‐specific health status of patients with SSc‐ILD. Additionally, the CAT may be useful for SSc‐ILD patients to determine effective strategies against pulmonary symptoms. For example, exercise training improved the 6MWD, and Medical Research Council scale and Chronic Respiratory Disease Questionnaire scores for interstitial lung disease (ILD) patients, including those with idiopathic pulmonary fibrosis.^18^

However, as the difference between IIP and SSc‐ILD in this study, CAT scores in IIP patients were significantly poorer in the presence of corticosteroids. There is no evidence of the efficacy of corticosteroids against idiopathic pulmonary fibrosis,^19^ but patients with IIP are recommended to receive corticosteroids if the symptoms are severe, that is, if they are in a poor condition.^20^ In contrast, the therapeutic target of corticosteroids for SSc patients is not limited to the lung symptom, they are also used for the treatment of diffuse skin disease, myositis and musculoskeletal manifestations.^11^, ^21^ Thus, the CAT score was considered statistically unaffected by the medication in this study.

A previous study reported that if patients with SSc did not develop ILD or PAH, the 6MWD was stable without deterioration for at least 66 months.^22^ This suggests that most of the deterioration in the 6MWD in patients with SSc‐ILD in this study was the outcome of PAH. SSc‐ILD patients with PAH, that develops in 7% of SSc cases, are known to have the poorer prognosis even compared with patients with idiopathic PAH.^11^ Related factors according to the Short Form‐36 physical component summary in SSc were reported to be age, diffuse cutaneous disease subtype and PAH.^23^ Little is known about the health status in PAH, but its progression in SSc‐ILD may also negatively influence the CAT score, as noted in this study.

The CAT score response to home oxygen therapy was similar between IIP and SSc‐ILD, and poorer scores were observed. However, as we did not compare the same patients by the CAT between with and without home oxygen therapy, it was difficult to determine if the poorer score was a direct result of supplemental oxygen. A previous study using the Short Form‐36 revealed that the negative effects of oxygen therapy in ILD patients, and self‐consciousness, inconvenience and cost of supplemental oxygen were reported to reduce the quality of life.^19^ Additionally, the study suggested the symptomatic benefit was thought to be smaller in ILD than in COPD.

The time since diagnosis, in this study, was significantly longer in SSc‐ILD patients than in those with IIP even though the CAT score was lower in SSc‐ILD patients. Recently, patients with IIP with autoimmune features, including SSc‐related autoantibody, were reported to have a poorer survival outcome than those with SSc‐ILD,^16^ but there was no difference in the survival rate from idiopathic pulmonary fibrosis.^24^ The longer survival duration in SSc‐ILD caused the longer duration until deterioration in the CAT score. A correlation between CAT scores and time since diagnosis was also found for SSc‐ILD in this study, which may have been due to the longer survival, as described above. The rapid pulmonary involvement in IIP may make it difficult to demonstrate a correlation with CAT scores within the short disease duration.

Our study demonstrated similarities and differences in the CAT between IIP and SSc‐ILD. Many accompanying symptoms exist in each CTD subgroup such as PAH in SSc. In general, many CTD patients exhibit arthritis and muscle weakness, which likely affect exercise capacity in addition to pulmonary impairment. For example, rheumatoid arthritis‐ILD, which symptomatically develops in approximately 10% of patients with rheumatoid arthritis, was identified as the main cause of morbidity and mortality.^25^ However, rheumatoid arthritis impairs multiple joints and it may be difficult to distinguish the effects of ILD on the quality of life. Moreover, in patients with idiopathic inflammatory myopathies featuring proximal muscle weakness,^26^ the dynamic repetitive muscle function was reported to affect the quality of life more than muscle strength as evaluated by the Manual Muscle Test.^27^ Thus, musculoskeletal limitation, if present, should be considered as an effector during evaluation with the CAT in further studies.

### 3.1 Limitations

This study was performed at a single facility, and IIP and SSc‐ILD were assessed as one disease group. However, it is known that there are many subtypes of SSc involving different organs with varying courses.^11^ Recently, positive autoantibodies in ILD have been utilised as clinical classification criteria,^28^, ^29^ which may provide more detailed features of the quality of life of patients if large cohorts are examined.

## 4 CONCLUSIONS

The CAT is a simple and reliable tool to assess the lung‐specific health status in patients with SSc‐ILD as IIP in terms of pulmonary function and distance walked, regardless of the differences in gender distribution and disease progression. Moreover, when SSc‐ILD is accompanied by PAH, the CAT score may be poorer.

## CONFLICT OF INTEREST

The authors declare that they have no competing interests.

## AUTHOR CONTRIBUTIONS

Designed the study and collected the data: All authors

*Analyzed the data*: Someya

*Wrote the paper*: All authors

## ETHICS

This study was approved by the Human Ethics Committee of Kanazawa University conforming to the provisions of the Declaration of Helsinki.
